# Genome-wide Target Enrichment-aided Chip Design: a 66 K SNP Chip for Cashmere Goat

**DOI:** 10.1038/s41598-017-09285-z

**Published:** 2017-08-17

**Authors:** Xian Qiao, Rui Su, Yang Wang, Ruijun Wang, Ting Yang, Xiaokai Li, Wei Chen, Shiyang He, Yu Jiang, Qiwu Xu, Wenting Wan, Yaolei Zhang, Wenguang Zhang, Jiang Chen, Bin Liu, Xin Liu, Yixing Fan, Duoyuan Chen, Huaizhi Jiang, Dongming Fang, Zhihong Liu, Xiaowen Wang, Yanjun Zhang, Danqing Mao, Zhiying Wang, Ran Di, Qianjun Zhao, Tao Zhong, Huanming Yang, Jian Wang, Wen Wang, Yang Dong, Xiaoli Chen, Xun Xu, Jinquan Li

**Affiliations:** 1 College of Animal Science, Inner Mongolia Agricultural University, Hohhot, Inner Mongolia, 010018 China; 2 Key Laboratory of Animal Genetics, Breeding and Reproduction, Inner Mongolia Autonomous Region, Hohhot, 010018 China; 3 Key Laboratory of Mutton Sheep Genetics and Breeding, Ministry of Agriculture, Hohhot, 010018 China; 4 Engineering Research Center for Goat Genetics and Breeding, Inner Mongolia Autonomous Region, Hohhot, 010018 China; 5 State Key Laboratory of Genetic Resources and Evolution, Kunming Institute of Zoology, Chinese Academy of Sciences, Kunming, 650223 China; 6 China National GeneBank, BGI-Shenzhen, Shenzhen, 518120 China; 7 BGI-Shenzhen, Shenzhen, 518083 China; 8 BGI-Qingdao, Qingdao, 266000 China; 9 College of Animal Science and Technology, Northwest A&F University, Yangling, 712100 China; 10 College of Biological big data, Yunnan Agriculture University, Kunming, 650504 China; 11 Center for Ecological and Environmental Sciences, Key Laboratory for Space Bioscience & Biotechnology, Northwestern Polytechnical University, Xi’an, 710072 China; 12 Institute of Animal Husbandry, Academy of Agriculture and Stockbreeding Sciences, Hohhot, Inner Mongolia, 010030 China; 13 College of Animal Science and Technology, Jilin Agricultural University, Changchun, 130118 China; 14 The Key Laboratory for Farm Animal Genetic Resources and Utilization of Ministry of Agriculture of China, Institute of Animal Science (IAS), Chinese Academy of Agricultural Sciences (CAAS), Beijing, 100193 China; 15 Institute of Animal Genetics and Breeding, Sichuan Agricultural University, Chengdu, 611130 China; 16 James D. Watson Institute of Genome Sciences, Hangzhou, 310058 China; 17 Yunnan Research Institute for Local Plateau Agriculture and Industry, Kunming, 650201 China

## Abstract

Compared with the commercially available single nucleotide polymorphism (SNP) chip based on the Bead Chip technology, the solution hybrid selection (SHS)-based target enrichment SNP chip is not only design-flexible, but also cost-effective for genotype sequencing. In this study, we propose to design an animal SNP chip using the SHS-based target enrichment strategy for the first time. As an update to the international collaboration on goat research, a 66 K SNP chip for cashmere goat was created from the whole-genome sequencing data of 73 individuals. Verification of this 66 K SNP chip with the whole-genome sequencing data of 436 cashmere goats showed that the SNP call rates was between 95.3% and 99.8%. The average sequencing depth for target SNPs were 40X. The capture regions were shown to be 200 bp that flank target SNPs. This chip was further tested in a genome-wide association analysis of cashmere fineness (fiber diameter). Several top hit loci were found marginally associated with signaling pathways involved in hair growth. These results demonstrate that the 66 K SNP chip is a useful tool in the genomic analyses of cashmere goats. The successful chip design shows that the SHS-based target enrichment strategy could be applied to SNP chip design in other species.

## Introduction

The first-generation animal breeding strategies for important quantitative traits relied heavily on keeping meticulous documentation of animal phenotypes and breeding values over several generations^1^. Because this process was expensive and time consuming^2^, breeding scientists tried to find more efficient methods to select desirable genetic traits. Nearly 35 years ago, Geldermann proposed the concept of quantitative trait loci (QTL) in animal breeding, which assumed that genes with related functions were often clustered in the genome to control biological traits^3^. Since then, the QTLs in association with genes or chromosome segments of interest have been widely studied to delineate complex animal traits^4–6^. In addition, marker-assisted selection (MAS)^7^ was devised to introduce desirable QTLs into an animal population or increase the proportion of desirable QTLs in the gene pool. Despite its usefulness, the MAS program used a small number of DNA markers to trace limited numbers of QTLs^8^. This disadvantage lead to the development of genomic selection (GS), which aimed to use simultaneously all available genome-wide dense SNP markers to predict breeding values^9^. Another strategy known as genome-wide association (GWA) analysis was also proposed, which believed that specific SNP markers could be in genetic linkage disequilibrium with a causative mutation affecting animal traits^10^. Therefore, identification of these significant genome-wide SNP markers is important for studying complex traits.

**Figure 1:**
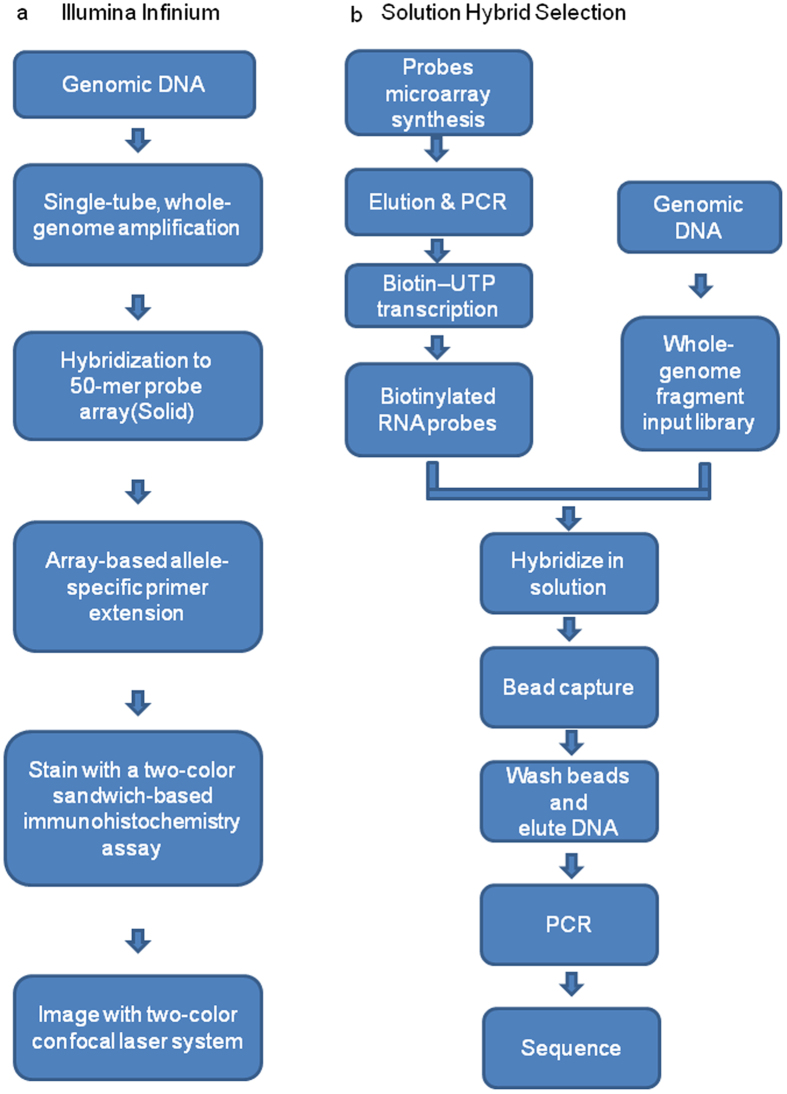
Schematic comparison between Illumina Infinium assay and solution hybrid selection (SHS)-based targeted sequencing method. (**a**) The schematic diagram of Illumina Infinium assay. (**b**) The schematic diagram of SHS-based targeted sequencing method.

Thanks to the development of next-generation sequencing technology, the cost of large-scale genotyping has been reduced dramatically. This technological advancement provides the possibility of designing and utilizing high-throughput SNP chip for animal breeding^11^. Among all available products on the market, the GoldenGate and Infinium analyses are extensively used in animal genetic studies. Both assays are based on Illumina’s Bead Chip technology, which involves direct hybridization of whole-genome amplified genomic DNA to a bead array of 50-mer locus-specific primers^12–14^, an enzymatic-based extension assay, a sandwich-based immunohistochemistry assay, and the final imaging by a two-color confocal laser system (Fig. 1a)^15^.

Target enrichment prior to sequencing is a useful method, in that specific portions of a genome can be analyzed to a greater depth^16^. This is due to the utilization of capture probes designed to select DNA regions of great interest. Compared with single locus genotyping, targeted sequencing can not only obtain a large scale of low density to high density SNPs, but also provide more information about the SNP variations, insertions/deletions, and copy number variations^17^. The major target enrichment strategies include molecular inversion probes^18^, SHS^19^, microarray-based GS^20^, and so on. Figure 1b shows the schematic steps of a SHS-based targeted sequencing process. Considering the sensitivity of genotyping, the uniform depth of coverage, and the scaling of reagent cost, SHS-based targeted sequencing is suitable for medium and large projects^21^.

Even though current commercial SNP chips based on the Bead Chip technology for different domestic animals have been successful^22–27^, we propose to design an animal SNP chip using the SHS-based target enrichment strategy for the first time. As an update to the international collaboration of goat research, we chose cashmere goat as our model animal. A 52 K SNP chip for goat (Illumina Inc., SanDiego, CA) developed by the International Goat Genome Consortium included sequencing data from Saanen, Alpine, Creole, Boer, Katjang, and Savanna goat breeds^28^. This chip has been widely used to study the genetic diversity^29^, population structure^30^, effective population size^31^, QTL detection^32^, and GS in multiple goat populations^33^. No special SNP chip is designed for cashmere goat till now.

Cashmere goat is a multipurpose breed that adapts well to the desert and semi-desert pastoral environments. This goat breed produces high-quality cashmere fiber, which is crucial to the world textile industry. It is estimated that cashmere goat herding has contributed substantial economic benefits to the local people in the remote regions of developing Asian countries^34, 35^, and the downstream industries have increased international trade between Asia and the developed world^36, 37^. The meat from free-ranging cashmere goat is also considered as delicacy, and has aroused the interest of many meat markets. Here, a moderate-density SNP chip for cashmere goat was designed using the SHS-based target enrichment strategy. This chip was subsequently tested in a population of Inner Mongolia cashmere goats, through which several potential loci related to cashmere goat traits were obtained through GWA analysis.

## Results

### 66 K SHS-based target enrichment SNP chip design for cashmere goat

**Figure 2:**
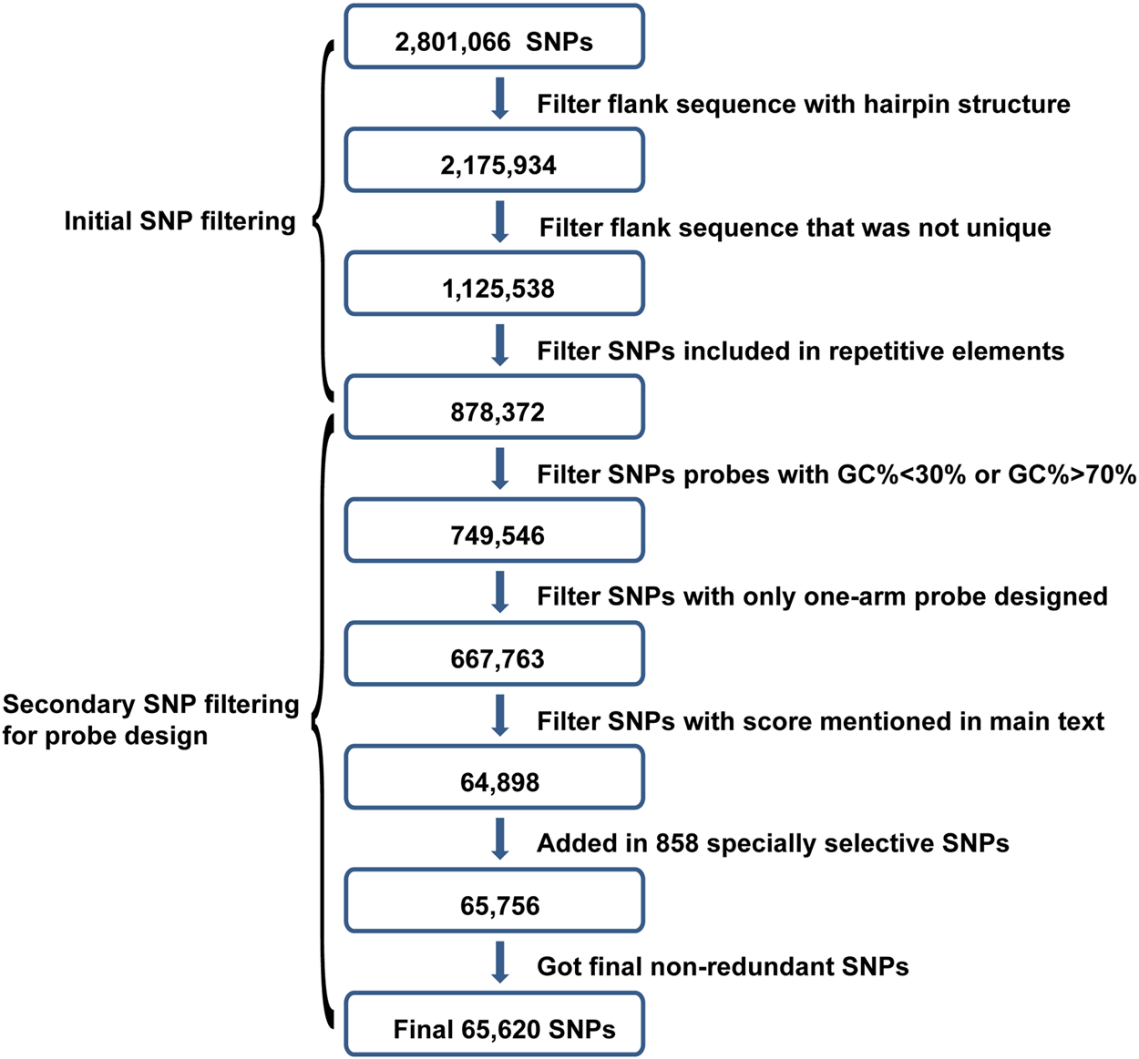
SNP selection and probe design workflow for the 66 K SNP chip.

**Figure 3:**
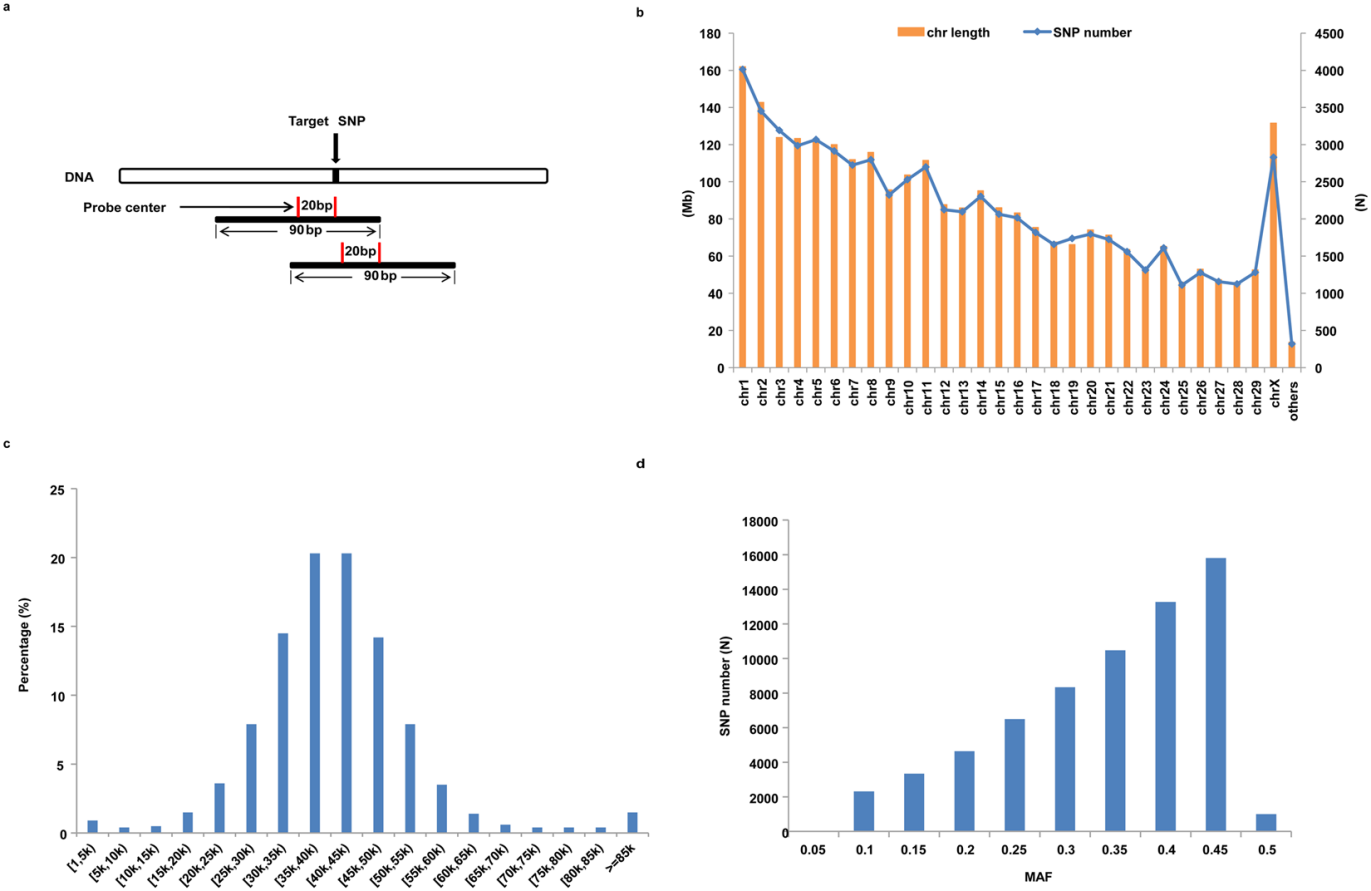
The characterization of final SNPs selected for designing the 66 K SNP chip. (**a**) The schematic graph of probe design. The distance between the center of probe and the target SNP is shown. (**b**) The number of SNPs corresponds to the lengths of scaffolds/chromosomes in the goat genome. (**c**) The frequency distribution of the spaces between neighboring SNPs. (**d**) The frequency distribution of the MAFs of the selected SNPs.

A total of 2,801,066 SNPs were called from the genome sequencing data of 73 cashmere goats (Fig. 2, Supplementary Table 1). These SNPs were used as candidates for SNP selection. After the first three steps of initial data filtering, 878,372 SNPs were obtained for the probe design process (Fig. 3a). The secondary filtering process of the designed probes yielded 64,898 SNPs from the cashmere goat population for the SNP chip. At this step, we decided to add 858 SNPs (courtesy of Dr. Jiang, data from 17 cashmere goats and 21 non-cashmere goats) in some genes related to wool fiber traits to our SNP pool. After the removal of redundant SNPs, a set of 65,620 SNPs were chosen for the chip design (Supplementary Table 2).

These SNPs spread over 30 chromosomes and 27 scaffolds (Fig. 3b). The frequency distribution of the spacing between adjacent SNPs revealed that about 95.7% of the SNPs were within 15 kb–70 kb to their closest neighbor counterparts (Fig. 3c), indicating no selection bias in the SNP-dense genomic regions. Additionally, the majority of the selected SNPs had a minor allele frequency (MAF) score greater than 0.2 (Fig. 3d). Based on these SNPs and their probe designs, a 188 K probe library was synthesized and separated in two 94 K sub-libraries. This marked the creation of a 66 K SHS-based target enrichment SNP chip for cashmere goat (Supplementary Table 3).

### Population of cashmere goats for GWA analysis

The average measurements of the diameters of cashmere fibers from 1,438 cashmere goats were ranked from the smallest value to the largest value with a range from 12.9 μm to 18.8 μm. At the population level, the diameter of cashmere fiber conformed to a normal distribution with 22 intervals (Supplementary Figure, K-S test, P = 0.174). We selected 300 cashmere goats from each of the two tails of this distribution, so that the average diameters of cashmere fibers had the largest difference between the two subpopulations (14.9 μm vs. 16.2 μm, Supplementary Table 4). After the removal of 164 cashmere goats without whole blood samples, a group of 436 cashmere goats (213 with smaller fiber diameter, 223 with bigger fiber diameter) were used for GWA analysis to test the 66 K SNP chip.

### SNP detection from 436 cashmere goats using the 66 K SNP chip

The 66 K SNP chip was used for the targeted sequencing of 436 cashmere goat genomes. About 15 Gb 50 bp single-end high-quality reads was produced for each animal on the BGISEQ-1000 platform. After initial data processing and population SNP calling, a total of ~407 K SNPs were obtained from these 436 animals. All sequencing reads covered about 95.3–99.8% of target SNPs on the chip depending on the individual goat.

In order to test the accuracy of SNP detection on different sequencing platforms, 11 target-enriched DNA sequencing libraries were randomly chosen from 436 samples and sequenced on an Hiseq. 2000 platform. These 11 libraries produced 46,395 SNPs from BGISEQ-1000, and 50,063 SNPs from Hiseq. 2000, respectively. The number of shared SNPs was 45,601, which accounted for more than 90% of detected SNPs on both platforms. This suggests that the 66 K SNP chip may be suitable for different sequencing platforms.

**Figure 4:**
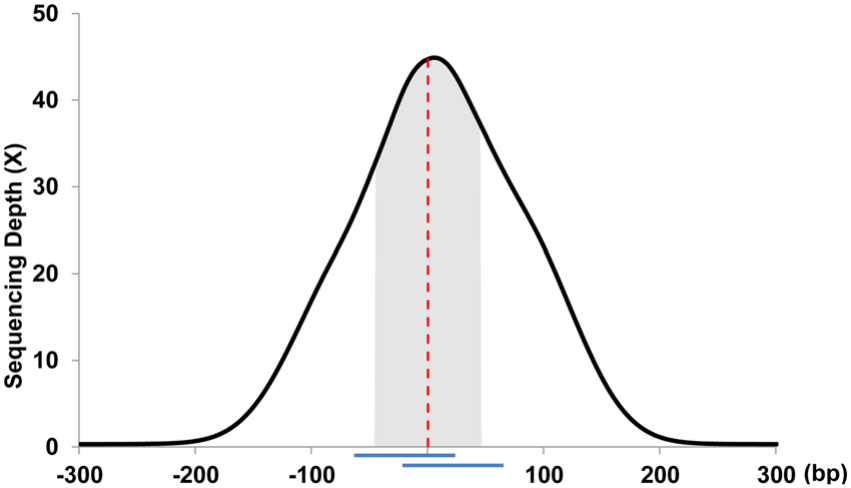
The sequencing depths of target SNP and its flanking regions. The curve is the base-by-base sequencing coverage around the target SNP. The probes are marked as the horizontal blue bars. The red dashed line in 0 position indicated the target SNP location.

The analysis showed that about 87% reads could be aligned to the reference genome, which included 56% reads within 300 bp from the target SNPs (Table 1). The estimation of sequencing depths of target SNPs and their 300 bp flanking regions showed a unimodal, center-weighted coverage distribution (Fig. 4). The majority of sequencing reads were located around target SNPs. The average sequencing depth of the target SNPs was 40X (Table 2). The average sequencing depth of the flanking regions decreased gradually as they distanced from the target SNPs. There were almost no reads covering regions 200 bp away from the target SNPs (depth ~2X). These results indicate that our 66 K SNP chip could effectively enrich the target SNP regions and narrow the flanking regions.

**Table 1:** Detailed description of sequences generated from catches.

List	amount or percentage
Aggregate length of target flanking region	19 Mb
Aggregate length of baits	11 Mb
Total raw unfiltered sequence	2,100 Mb
Aligned to reference genome	1,849 Mb (87%)
Uniquely aligned to reference genome	1,666 Mb (78%)
Aligned to flanking region	1,149 Mb (56%)
Aligned to off target region	700 Mb (30%)
Target SNP coverage	95.3–99.8%

**Table 2:** Detailed depth description of different type of position.

**List**	**before filter (fraction)**
MAF (<0.05) in 65 K target SNP	109 (<0.2%)
Average sample missing in 65 K target SNP	5 (1.26%)
Depth in 65 K target SNP	~40X
Depth in 200 bp flanking region	~20X
Depth not in target capture region	~2X

**Figure 5:**
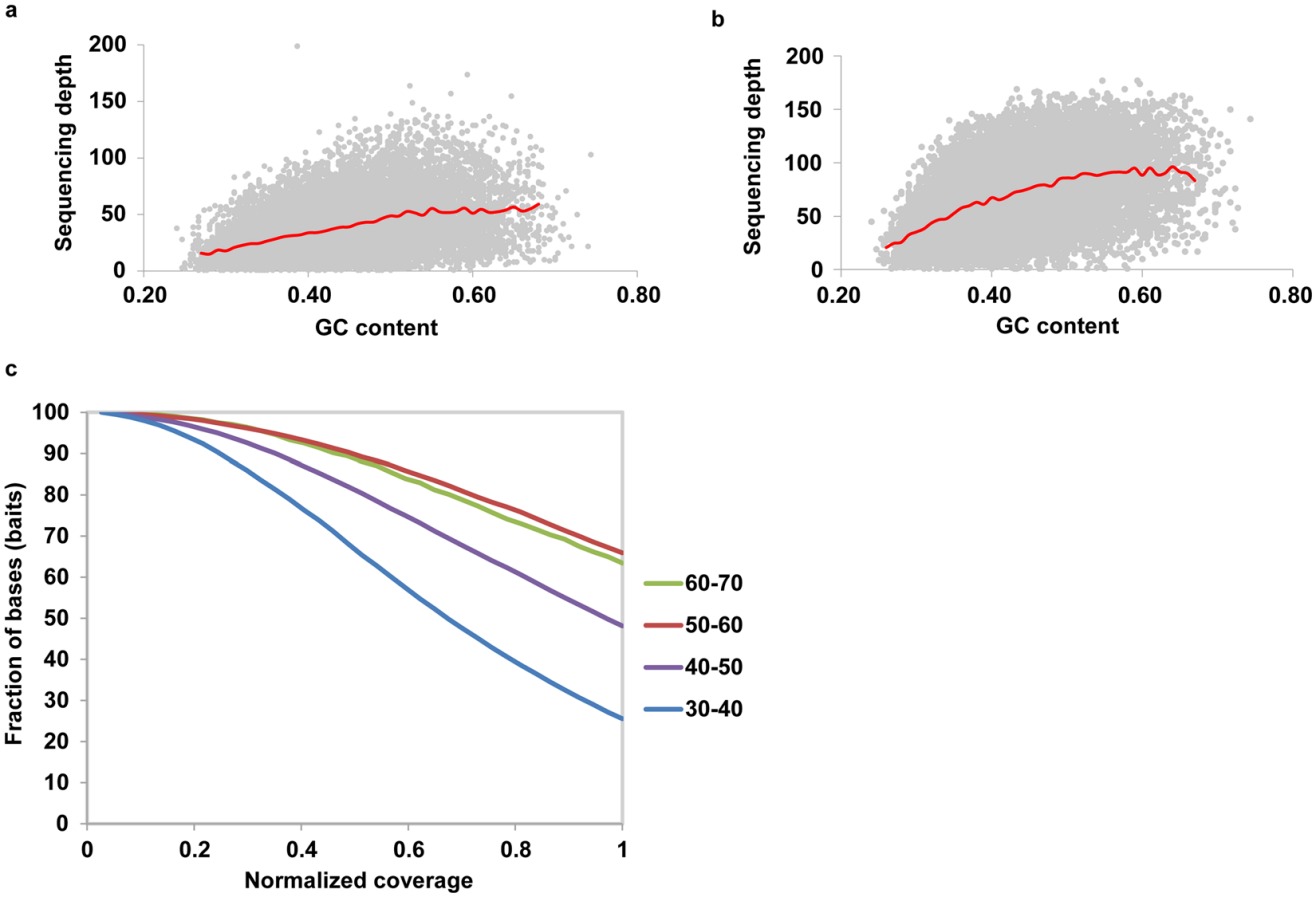
The relationship between GC content and sequencing depths. (**a**) Average depth of target SNP was 37. (**b**) Average depth of target SNP was 69. The red lines indicated mean depth. (**c**) Normalized coverage distribution plots in four different GC% categories.

It has been reported that many factors during sequencing steps, including polymerase chain reaction^38^, size-selection^39^, and probability of sequencing-errors^40^, could cause GC-content bias. To inspect the effect of sequencing depths on GC bias, GC contents were compared against mean read depths across all capture regions, with an average depth of 37 and 69, respectively (Fig. 5a,b). In addition, the genomic regions with 50–70% GC content had higher coverage and higher depth. In comparison, those genomic regions with 30–50% GC content had relative lower coverage and depth (Fig. 5c).

**Figure 6:**
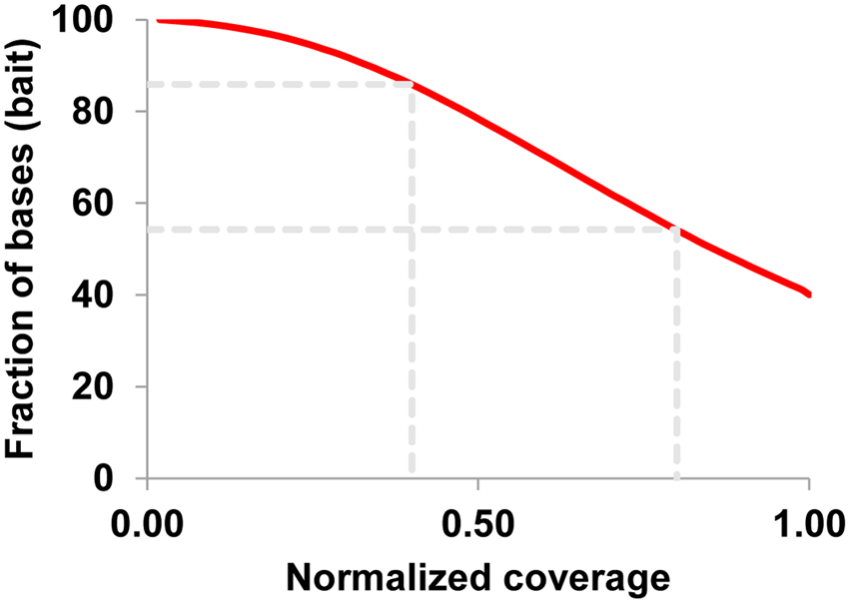
Normalized coverage distribution plots.

Uniformity of coverage is another important parameter for targeted sequencing. The coverage of each target SNP was normalized to the mean coverage observed across the entire set (Fig. 6). Plot showed that 54% of the target SNPs had at least 80% of mean coverage, and 86% of the target SNPs had at least 40% of mean coverage.

### SNP selection for GWA analysis

A total of 5,501,922 raw SNPs were obtained, 5,131,533 SNP with call rates <0.90. 13 animals with genotypes call rates <0.90, after removing non-biallelic SNPs (singleton/multi-allelic SNP), 423 cashmere goats with 370,389 SNP were left. The MAF and Hardy-Weinberg equilibrium (HWE) screening resulted in 161,125 SNPs for the following analyses. Among these, 55,863 SNPs matched the target SNP loci on the 66 K SNP chip.

### Population structure analysis

**Figure 7:**
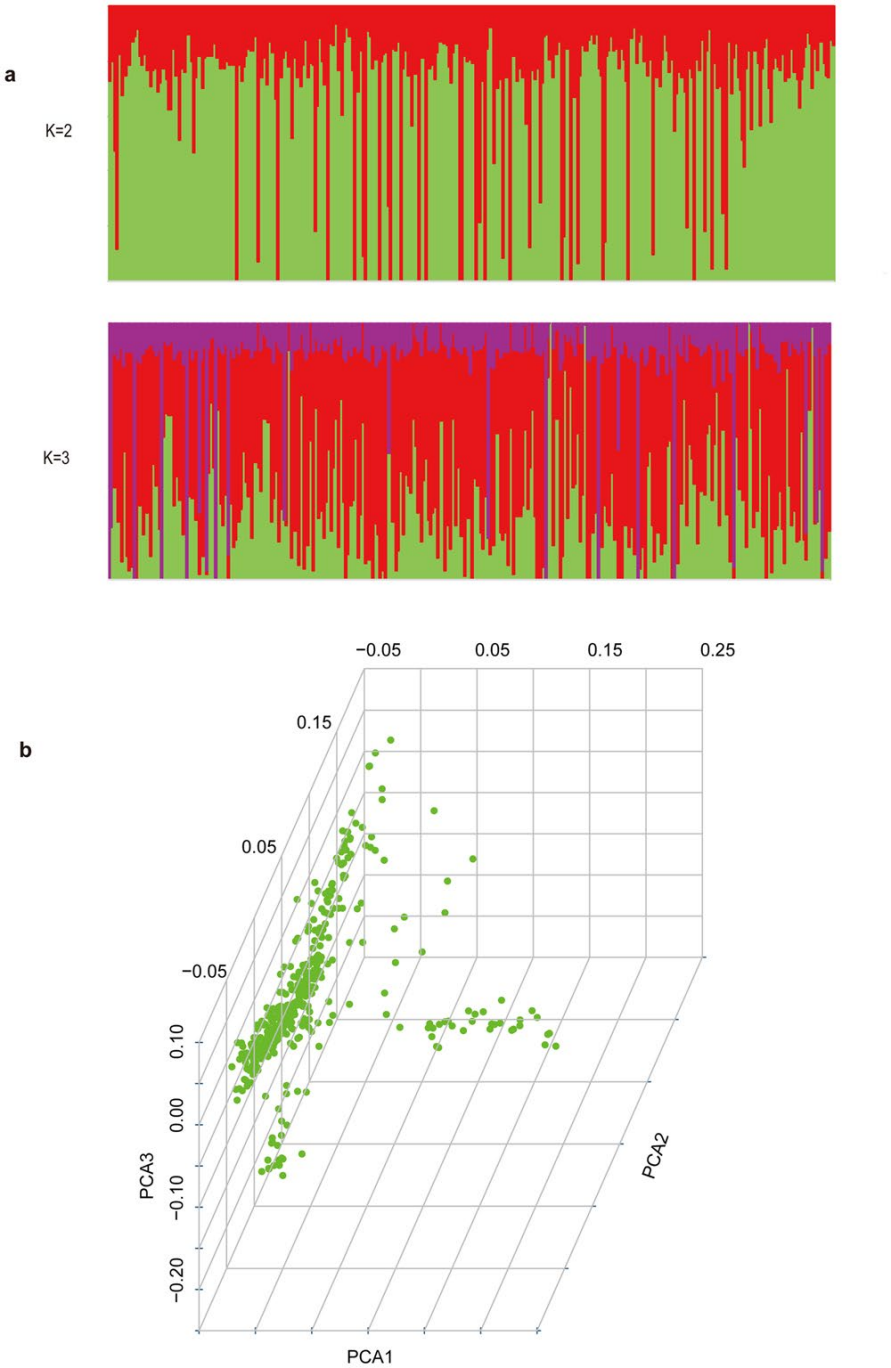
Population structure analysis of Inner Mongolia cashmere goat (Erlangshan). (**a**) Population structure analysis using STRUCTURE. Each sample is represented by a vertical bar. Enery color represents one ancestral population and the lenth of each colored seqment in each vertical bar represents the proportion contributed by ancestral populations. (**b**) PCA using all identified SNPs as markers. Each dots are index to samples, most samples cluster together.

We used PLINK software to conduct the population stratification analysis based on pairwise IBS distance, and found all the 423 samples were clumped into the same cluster. We ran STRUCTURE to determine the genetic ancestry constituents of samples and found that all samples almost have the same mixed ancestry when K = 2 or K = 3 (Fig. 7a). The results of principal component analysis (PCA) analysis showed that three principal components (PC1, PC2 and PC3) did not divide the populations (Fig. 7b).

### GWA analysis for cashmere fiber trait

**Figure 8:**
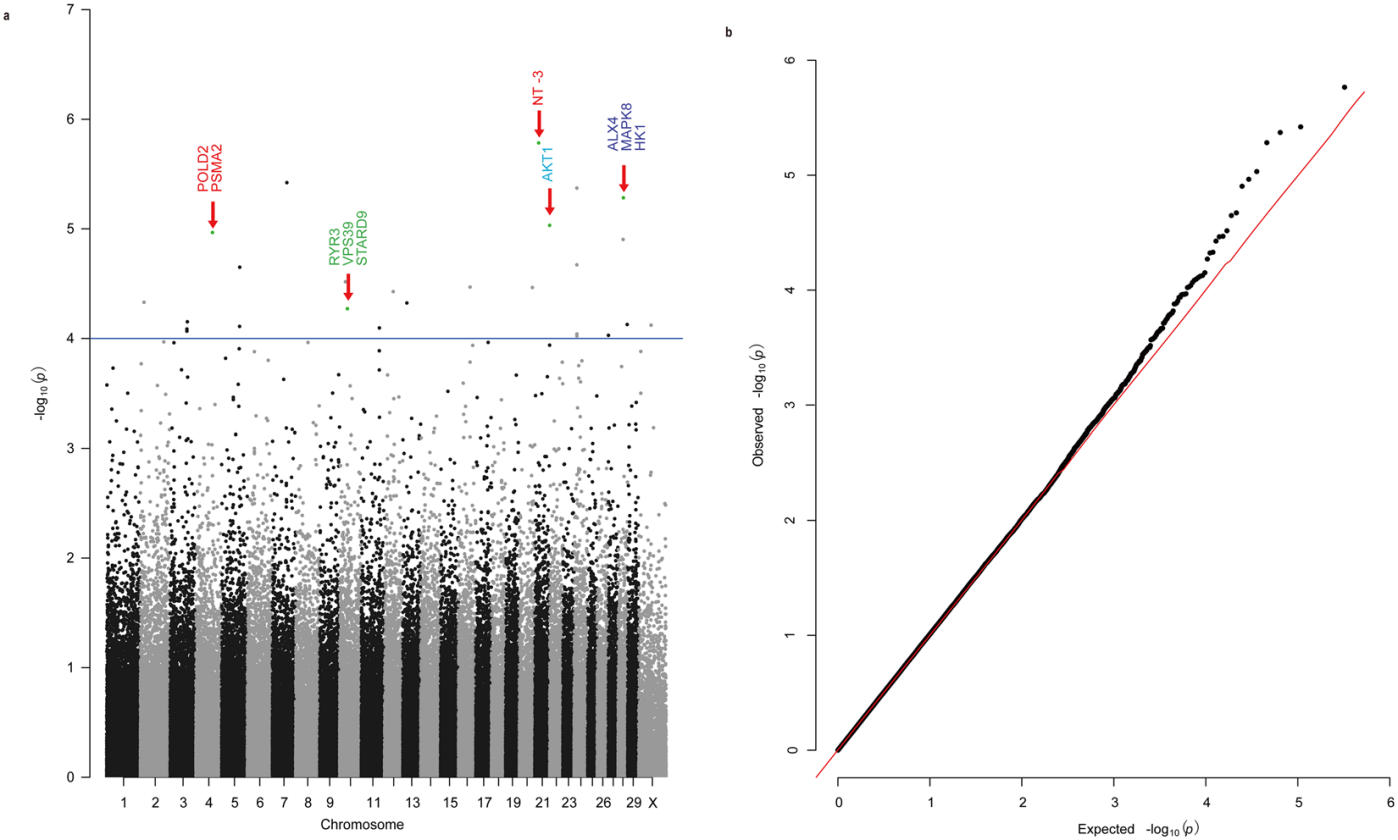
Manhattan and quantile-quantile plot of GWAS for fiber fineness in cashmere goat. (**a**) Manhattan plot of the compressed MLM model for fiber fineness. (**b**) Quantile-quantile plot of the compressed MLM model for fiber fineness.

The cashmere fiber quality is a complex quantitative trait related to several parameters, including fiber length, guard hair length, fiber fineness (diameter), and combed cashmere fiber weight^41^. In this study, 423 goat individuals were subjected to quantitative trait association analysis. As shown in the Manhattan plot, no association were observed between SNPs and fiber fineness at the genome-wide level statistical significance (P*-*value < 4.2 × 10^−7^, Fig. 8a), However, several peaks were observed with marginal statistical significance. This result corresponded to the hypothesis that the genetic control of fiber fineness involves multiple QTLs of minor effects^42^. No genomic inflation was observed according to the quantile-quantile plots with λ value of 1.013 (Fig. 8b).

To extract some useful information from this analysis, 26 top-hit loci were chosen for the annotation against the KEGG database (http://www.kegg.jp/) (Supplementary Table 5). The result showed that genes with some top-hit SNPs are involved in signal pathways that are associated with the development of hair follicles (e.g. 4 enriched in MAPK, 2 in Wnt, 2 in TGF and 1 in Notch and so on)^43^. Some other genes with top-hit SNPs were reported to be important for skin or hair follicle growth and development (Table 3).

**Table 3:** Biological inferences analysis of top-hit SNP.

ID	Symbol name	P value	Signal Pathway	Type	Reference
K04456	AKT1	9.29E-06	MAPK&Notch	Hair follicle	Dipoï, N. *et al*.^49^
					Yang, Z. Z. *et al*.^50^
					Mauro, T. M. *et al*.^51^
K09451	ALX4	7.46E-05	WNT&MAPK	Hair follicle	Kayserili, H. *et al*.^52^
					Boras, K. *et al*.^55^.
K00844	HK1	5.21E-06	TGF&Shh	Hair follicle	Ellis, T. S. I. *et al*.3^57^.
K05101	NT-3 growth factor receptor	1.72E-06		Hair follicle	Botchkarev, V. A. *et al*.^58^.
K02328	POLD2	1.08E-05	WNT	Skin	Baldeck, N. *et al*.^59^
K02726	PSMA2	1.08E-05	WNT	Skin	Park *et al*.^60^.
K04440	MAPK8 (JNKs)	7.46E-05	WNT&MAPK	Skin	Schumacher, M. *et al*.^61^.
K04963	RYR3	3.04E-05	TGF&MAPK	Skin	Denda, S. *et al*.^62^.
K20183	VPS39 (TLP)	5.36E-05	MAPK	Skin	Wang, X. *et al*.^63^
K16491	STARD9	5.36E-05	MAPK	Skin	Lin. *et al*.^64^

## Discussion

### SNP genotyping by target enrichment

Compared with other genotyping methods, SNP genotyping by target enrichment is a cost-effective method. Take the 66 K SHS-based target enrichment SNP chip for cashmere goat as an example, the cost was estimated to be about 214 dollars per sample for an expected sequencing depth of 10X and 138 dollars per sample for sequencing depth of 6X. Despite the low price, this chip can capture regions up to 200 bp away from target SNPs. It also shows that the sequencing depth was highest for the target SNP, suggesting a high efficiency for target enrichment. This feature helps customize the amount of sequencing data according to user’s need. For instance, the center of the SNP probes in this study was about 20 bp away from the target SNP. If a sequencing depth of 20X for target loci was expected, about 8 kb sequencing data would be sufficient for covering target regions. The required data would decrease to even lower amount, if the capture region is of a narrower scale. Another prominent advantage of this method is the flexibility showed in the chip design. The potential number of SNPs is not limited to a certain number. More SNP loci could be added to the chip for extra costs. This suggests that the target enrichment-based SNP chips can be designed for other species in the future, and be used for larger and more complex research projects.

### Comparison of the 66 K SHS-based target enrichment SNP chip for cashmere goat with the 52 K SNP Bead Chip for goat

The SNPs on the 52 K Bead Chip were identified from a total of 97 animals of six goat breeds, without cashmere goat. This chip was used to investigate the genetic diversities and populations structures of Italian goats and South African goats, respectively^29, 30^. The result of the first study indicated that the genetic diversities of the present-day Italian goat populations were shaped by the combined effects of drift, gene flow, and recent demographic history^29^. The author of the second study, using a subset of 44,660 SNPs from 216 individuals, argued that the indigenous South African goats had a high genetic diversity^30^. The 66 K SHS-based target enrichment SNP chip, in comparison, is a suitable genomic research tool for cashmere goat. This chip was designed with extensive sample sources, especially Chinese cashmere goat breeds. It is therefore very useful for population genetic analysis.

Lashmar *et al*.^44^ attempted to use the 52 K SNP Bead Chip to investigate genetic markers that are associated with fiber-producing traits in goat breeds. It is worth noting that only 10,659 out of the total 53,347 SNPs could be used for association analyses. In addition, both Becker *et al*.^45^ and Martin *et al*.^46^ used the 52 K chip to find SNPs associated with the coat color trait in the Coppernecked goats and Saanen goats, respectively. The results of all of these studies were nevertheless not very ideal. In comparison, about 161,125 valid SNPs were captured from 436 cashmere goats with our 66 K chip for subsequent GWA analyses, showing a much potent way of acquiring large genetic information. Even though no statistically significant genetic contributors to cashmere fineness were identified in the GWA analyses, some top-hit SNPs seemed to be associated with physiological pathways that are important for hair development. One possible explanation for this result might be the moderate phenotypic variance in cashmere fineness (14.9μm vs. 16.2μm) in the cashmere goat populations. Another possible explanation might be that cashmere fineness is controlled by a combination of genetic and environmental factors.

It is now a common practice to use genome-wide SNPs generated by whole-genome sequencing or high-density SNP chip to carry out animal genetics research. It is not limited to traditional genomics research, but more and more used in studies on interesting animal traits. For example, Yang *et al*. showed the impact of global climate change on native sheep rapid adaptations to extreme environments. Kijas *et al*. and Lv *et al*. used Ovine SNP50K Beadchip to study the genetic history of global sheep breeds^47^, the adaptation to climate-mediated selection^48^, and so on. These successful application cases showed the great potential of genome-wide SNPs and high-density SNP chip technology. Even though our chip is most suitable for analyzing cashmere fiber trait, it is worth mentioning that it can be modified to study other cashmere goat traits, and used in animal breeding. Our chip design method can also be exploited for other species in the future.

### The top-hit SNPs associated with fineness

The result of KEGG analysis discovered some top-hit SNPs near the genes, which are involved in hair follicle development. For example, *AKT1* gene belongs to the Notch and MAPK signal pathway (Table 3). It has been reported that *AKT1* activity is required for hair peg elongation during the hair follicle development. The hair follicles were distinctly reduced in size in *Akt* ^+/−^ *Akt3* ^+/−^ animals compared with their wild counterparts^49–51^. Another gene, *ALX4*, plays a critical role in skin and hair follicle development in human^52^. *ALX4* binds to *LEF-1*, a key regulatory factor for hair follicle development^53, 54^, and regulates its N-CAM promoter activity. Both *LEF-1* and *Alx4* knockout animals have defects in the hair follicle development^55^. In addition, *HK1* gene regulates the Shh pathway in the hair follicle^56^ to inhibit the embryonic hair follicle morphogenesis^57^. Besides, the gene *NT-3* in the top-hit SNP locus (Fig. 8a) encodes a growth factor receptor, which is known to be important for hair follicle development^58^. Other genes around the top-hit SNPs are proved to be related of hair and skin^59–64^.

It can be concluded from the results that the breed-based application of our 66k goat SNP chip for cashmere fineness trait is possible. Even though no significant major singular contributor to fineness was included in our method, some top-hit SNPs proved to be important to hair follicle observed in this study by a medium density chip. It suggests that the 66k goat SNP chip will allow for applications such as GWAS, diversity studies, selection signatures and eventually genomic selection in the future.

## Methods

### Cashmere goat populations used in this study

One group of 73 female cashmere goats sampled from four pastoral locations in Inner Mongolia and Liaoning Provinces (19 Erlangshan, 16 Alashan, 19 Albas, and 19 Liaoning) were used in the extraction of SNP variants and the subsequent design of a 66k SNP chip. A second group of 1,438 female goats from the Tonghetai Breeding Farm in Erlangshan were used in the testing of the designed SNP chip with GWA analysis (Supplementary Table 1). All goats used in this study were raised in the free-ranging style.

### Venous whole blood sample collection and genomic DNA extraction

With the assistance of local herdsmen, trained veterinarians randomly chose three-year-old female cashmere goats from the populations, and collected 5 ml whole blood from the left jugular vein of each animal into a plastic collection tube containing 4% (w/v) sodium citrate. The blood samples were snap frozen in liquid nitrogen, and stored at −80 °C until further processing. Genomic DNA was extracted from whole blood samples with the AXYGEN Blood and Tissue Extraction Kit (Corning, USA) according to the manufacturer’s instructions. The extracted DNA was subjected to electrophoresis in 2% agarose gel and stained with ethidium bromide to assess overall quality. The DNA concentration was determined by Quant-iT™ PicoGreen ® dsDNA Reagent and Kits (Thermo Fisher Scientific, USA) according to the manufacturer’s instructions. All animal procedures were approved by the Inner Mongolia Agriculture University Animal Care and Use Committee in accordance with the National Animal Care Standard (GB 14925–2001). All experiments were performed in accordance with relevant guidelines and regulations. All efforts were made to minimize animal suffering.

### Chip design: library construction, sequencing, SNP discovery and characterization

For the group of 73 cashmere goats, paired-end libraries were constructed for each individual animal with an insert size of 300 bp from ~2 μg of sheared genomic DNA according to the procedures of NEB DNA Library Prep Kit for Illumina (NEB, USA). These libraries were sequenced on an Illumina Hiseq. 4000 platform (Illumina; CA, USA) using a PE-100 module. After data filtering, high quality reads were mapped to the goat reference genome (version 2.0)^65^ using the Burrows-Wheelser Aligner (version 0.7.10-r789)^66^ with default settings. The software SAMtools^67^ was used to convert file format from SAM to BAM. The package Picard (http://broadinstitute.github.io/picard/) was used to sort BAM files by coordinate and mark PCR duplications.

After the BWA alignment process, ‘RealignerTargetCreator’ and ‘IndelRealigner’ in the Genome Analysis Toolkit (GATK, version 3.3–0-g37228af)^68^ were used to obtain duplication-free reads with default settings. Next, ‘HaplotypeCaller’ in the GATK was used to generate a single call set from the sequencing data of all 73 individuals by joint calling with the parameter ‘-stand_call_conf 30 -stand_emit_conf 10’. The SNP data were then distinguished from the InDel data by ‘selectVariants’ in the GATK. The criteria^68^ used to exclude false positive SNP data were the following: (a) hard filtration with the parameter ‘QD < 2.0 || FS > 60.0 || MQ < 40.0 || MQRankSum <−12.5 || ReadPosRankSum <−8.0′; (b) total depth does not range from 80 to 1000; (c) missing data rate >20% and >20% individual depth <2.

### Chip design: SNP selection and probe design

In brief, the probe design process includes the selection of SNP-containing genomic regions and the optimization of the probe hybridization efficiency (Fig. 2). The goal is to cover the whole genome as much as possible while achieving a low off-target hybridization rate. To this end, SNPs were initially filtered according to the following criteria: (1) the flanking sequences within 150 bp on either side of a SNP should not form any >8 bp hairpin structure; (2) the flanking sequences on either side of a SNP should be unique; (3) Exclude any SNPs in the repetitive elements. The resultant SNPs were viewed as candidates for designing SNP probes.

A SNP probe is a 120 bp oligonucleotide, which contains a 90 bp target-specific bait sequence and two 15 bp PCR primer ends (5′-GAA GCG AGG ATC AAC [N90] CAT TGC GTG AAC CGA-3′). The 90 bp target-specific bait sequence pairs with the genomic region containing the target SNP. In this study, a series of probes were designed to cover each specific SNP with the stipulation that the center of the probe should be less than 50 bp away from the target SNP. These probes were screened by the GC content criterion (between 30% and 70%). SNPs with no viable probe designs or with only one viable probe design were removed from the candidate pool. If multiple probe designs were available for a SNP, two or three best probes were selected on the condition that the center of the probe should be about 20 bp away from the target SNP (Fig. 3a). For some SNPs, if one of the only two viable probe designs contains any >8 bp hairpin structure, the other probe will be kept and used twice the amount for the final chip. In addition, any probes that cover two SNPs will also be used twice the amount for the final chip.

In order to cover the goat genome on a 60–70 K SNP chip, the average interval between SNPs is estimated to be about 40 kb. We further optimized the target SNPs density in consecutive 40 kb genomic windows. A ranking score was calculated for each of the remaining SNPs according to the following formula^69^:

$$Score=MAF\,\ast \,(E-S)\,\ast \,(1-\frac{|a-(E-S)/2|}{(E-S)/2})$$

*MAF* is the minor allele frequency. *a* is the coordinate of a SNP. *S* and *E* are the initial coordinate, and stop coordinate of the 40 kb genomic window, respectively. In SNP-dense genomic regions, only those with high ranking scores were kept for the chip design.

### Chip design: SNP probe synthesis, processing, and the final chip

All final SNP probes were obtained with a CustomArray B3™ Synthesizer (CustomArray, Washington DC, USA) according to the manufacturer’s instructions. The probe libraries were aminolyzed, purified, and then dissolved in 10× TE buffer (pH = 8.0). The probe libraries were amplified by PCR with primers A (5′-GAA GCG AGG ATC AAC-3′) and B (5′-TCG GTT CAC GCA ATG-3′). With the addition of SP6 promoter sequences, the probe libraries were transcribed into RNA bait libraries with SP6 RNA polymerase. They were then labeled by biotin. After purification and quality control, biotinylated RNA bait libraries were prepared for hybridization and stored at −70 °C (Fig. 1b), which were the final product of the SHS-based target enrichment SNP chip.

### Cashmere fiber sample collection and analysis

For the group of 1,000 cashmere goats for GWA analysis, about one gram of cashmere fiber sample was obtained from the left scapular region of each individual animal. The average diameter of the cashmere fibers from each individual was assessed by an OFDA-2000 optical-based fiber diameter analyzer (BSC Electronics, Australia) according to the manufacturer’s instructions.

### Targeted sequencing of 436 cashmere goats

1 μg high-quality genomic DNA from each cashmere goat was subjected to sonication. The DNA fragments of 150–250 bp in lengths were selected for the targeted sequencing. These fragments were end-repaired before being ligated to the Ad153Ω_2B adapter (BGI Shenzhen, China, unpublished). They were then amplified by a round of standard PCR. The biotinylated RNA bait libraries (see methods above) were used to capture and enrich SNP-containing DNA fragments. Captured DNA fragments were subjected to a round of standard PCR, and the PCR products were circularized and made ready for sequencing (Fig. 1b) on a BGISEQ-1000 platform (BGI Shenzhen, China) with a SE50 module.

### Variant detection in the GWA population

Raw sequencing reads were filtered according to the following criteria: (1) if a read has >10 percent of bases as N; (2) if a read has >40 percent of low-quality (value <=10) bases; (3) if a read is contaminated by the adaptor sequence or produced by PCR duplication. The resultant clean reads were then mapped to the goat reference genome (v2.0) using BWA with parameters “-m 200000 -l 20 -k 2 -t 30”. The results were transformed into indexed BAM files with SAMtools (version 0.1.18). Picard package (version 1.105) was used to remove duplicate reads. Reads coverage and depth were calculated from BAM files with “samtools depth”. The variants were called using the Genome Analysis Toolkit’s HaplotypeCaller. After separating SNPs from Indel variants, SNPs was further filtered using the VariantFiltration package in GATK with parameters “–filterExpression “QD < 4.0 || MQ < 40.0 || ReadPosRankSum <−8.0 || FS > 60.0 || HaplotypeScore > 13.0 || MQRankSum <−12.5” –filterName LowQualFilter”.

### SNP data quality control

The following quality control process for our data was conducted by Plink v1.07 (http://pngu.mgh.harvard.edu/~purcell/plink/download.shtml) unless stated otherwise. Chromosomal variant cell format files were transformed into Plink format by VCFtools v0.1.13, during which the non-biallelic SNPs were automatically filtered out in the PED/MAP files. Because human (n = 23) was set as the default chromosome handling type as in Plink, the parameter*−dog* (n = 39) was added in front of each command line to ensure that the system could process all goat chromosomes (n = 30). SNPs with call rates <0.90 and samples with genotyping call rates <0.90 were removed for the further statistical analyses. The SNPs from the final goat individuals were subjected to quality control according to the following two criteria: (1) Remove SNPs with a very low MAF filtration (MAF < 0.01); (2) Remove SNPs with significant deviations from HWE filtration (HWE < 0.001).

### Population structure analysis

Population substructure was investigated using Clustering, STRUCTURE^70^ and PCA^71^ based on using genomic SNPs. We used Plink to do stratification analysis based on pairwise identity-by-state (IBS) distance with option–cluster–mc 2–ppc 0.05. Further STRUCTURE was used to infer genetic ancestry constituents and assign individuals to subpopulation. We also performed a PCA following the procedure as reported^71^. The eigenvector decomposition using the R function eigen, and the significance of the eigenvectors was determined with a Tracey-Widom test.

### Association analysis

The compressed mixed linear model(MLM) were used to identify association signals with the software EMMAX^72^. The basic model underlying this software can be written as

1 $${y}_{i}=\beta 0+\sum _{k=1}^{M}{\beta }_{k}{X}_{ik}+{\varepsilon }_{i}$$

2 $${\rm{V}}{\rm{a}}{\rm{r}}(Y)=2{\delta }_{ak}^{2}{\varphi }_{k}+2\varphi {\delta }_{a}^{2}+{\delta }_{e}^{2}I$$

In equation (1) the vector Y = {*y1*, …, *yn*} contain the phenotypes of the individuals, ${\rm{Var}}({\rm{\varepsilon }})={\delta }_{e}^{2}I,$ Var(*Y*) was used to investigate the contribution of locus k to the phenotype which the effect of the genotype at locus k can be modeled as a main effect, whereas the relationships among all individuals are taken into account by means of variance components of random polygenic effects. We calculated an identity-by-state kinship matrix using the Affymetrix genotypes in EMMAX with command “emmax-kin -v -h -s -d 10”, pairwise relatedness matrix was used to represent the sample structure. Using a variance component model, we got an estimated covariance matrix that models the effect of genetic relatedness on the phenotypes. Animal pasture information was used as covariate matrix. For cashmere trait, the threshold P-value for declaring genome-wide significance (*P* < 4.2 × 10^−7^) which was set to control genome-wide type 1 error rate. Manhattan plot was drawn by *qqman* package of R (v3.2.0). A 500 kb region on each side of peak SNP was searched for gene annotation.

## Electronic supplementary material

Supplemental Figure

Dataset 1

Dataset 2

Dataset 3

Dataset 4

Dataset 5

## References

[1] Lush JL (1947). Family Merit and Individual Merit as Bases for Selection. Part I. The American Naturalist.

[2] Schaeffer LR (2006). Strategy for applying genome-wide selection in dairy cattle. Journal of Animal Breeding and Genetics.

[3] Geldermann H (1975). Investigations on inheritance of quantitative characters in animals by gene markers I. Methods. Theoretical and Applied Genetics.

[4] Soller, M. The use of loci associated with quantitative effects in dairy cattle improvement. *Animal Production* **27** (1978).

[5] Smith C, Simpson SP (1986). The use of genetic polymorphisms in livestock improvement. Journal of Animal Breeding and Genetics.

[6] Dekkers JC, Hospital F (2002). The use of molecular genetics in the improvement of agricultural populations. Nature Reviews Genetics.

[7] Lande R, Thompson R (1990). Efficiency of marker-assisted selection in the improvement of quantitative traits. Genetics.

[8] Golding B, Hayes B, Goddard M (2010). Genome-wide association and genomic selection in animal breeding. Genome.

[9] Meuwissen TH, Hayes BJ, Goddard ME (2001). Prediction of total genetic value using genome-wide dense marker maps. Genetics.

[10] Visscher PM (2012). Five Years of GWAS Discovery. The American Journal of Human Genetics.

[11] Hui Zhang, Z. W., Wang, S. and Hui, Li. Progress of genome wide association study in domestic animals. *Journal of Animal Science and Biotechnology* **3** (2012). 10.1186/2049-1891-3-26 PMC3506437 22958308

[12] Gunderson KL, Steemers FJ, Lee G, Mendoza LG, Chee MS (2005). A genome-wide scalable SNP genotyping assay using microarray technology. Nature Genetics.

[13] Steemers FJ, Gunderson KL (2005). Illumina, Inc. Pharmacogenomics.

[14] Gunderson KL (2006). Whole-genome genotyping of haplotype tag single nucleotide polymorphisms. Pharmacogenomics.

[15] Steemers FJ, Gunderson KL (2007). Whole genome genotyping technologies on the BeadArray™ platform. Biotechnology Journal.

[16] Mertes F (2011). Targeted enrichment of genomic DNA regions for next-generation sequencing. Briefings in Functional Genomics.

[17] Li W (2015). Identifying Human Genome-Wide CNV, LOH and UPD by Targeted Sequencing of Selected Regions. PLoS ONE.

[18] Porreca GJ (2007). Multiplex amplification of large sets of human exons. Nature Methods.

[19] Gnirke A (2009). Solution hybrid selection with ultra-long oligonucleotides for massively parallel targeted sequencing. Nature Biotechnology.

[20] Albert TJ (2007). Direct selection of human genomic loci by microarray hybridization. Nature Methods.

[21] Teer JK (2010). Systematic comparison of three genomic enrichment methods for massively parallel DNA sequencing. Genome Research.

[22] Georges M (2012). A High Density SNP Array for the Domestic Horse and Extant Perissodactyla: Utility for Association Mapping, Genetic Diversity, and Phylogeny Studies. PLoS Genetics.

[23] Groenen, M. A. M. *et al*. The development and characterization of a 60K SNP chip for chicken. *BMC Genomics* **12** (2011). 10.1186/1471-2164-12-274 PMC3117858 21627800

[24] Orban L (2009). Design of a High Density SNP Genotyping Assay in the Pig Using SNPs Identified and Characterized by Next Generation Sequencing Technology. PLoS ONE.

[25] Lühken G (2012). Genetic testing for phenotype-causing variants in sheep and goats. Molecular and Cellular Probes.

[26] Harris, B. L., Creagh, F. E., Winkelman, A. M. & Johnson, D. L. Experiences with the Illumina High Density Bovine BeadChip. **44** (2011).

[27] Wiggans GR, Cooper TA, Van Tassell CP, Sonstegard TS, Simpson EB (2013). Technical note: Characteristics and use of the Illumina BovineLD and GeneSeek Genomic Profiler low-density bead chips for genomic evaluation1. Journal of Dairy Science.

[28] Liu Z (2014). Design and Characterization of a 52K SNP Chip for Goats. PLoS ONE.

[29] Nicoloso, L. *et al*. Genetic diversity of Italian goat breeds assessed with a medium-density SNP chip. *Genetics Selection Evolution* **47** (2015). 10.1186/s12711-015-0140-6 PMC4523021 26239391

[30] Mdladla K, Dzomba EF, Huson HJ, Muchadeyi FC (2016). Population genomic structure and linkage disequilibrium analysis of South African goat breeds using genome-wide SNP data. Animal Genetics.

[31] Negrini, R. I E Past Population Size Changes of Italian Goat Breeds. *Plant & Animal Genome* (2014).

[32] Palhire, I., Larroque, H., Virginie, C., Tosser-Klopp, G. & Rachel, R. Genetic Parameters and QTL Detection for Milking Speed in Dairy Alpine and Saanen Goats. *World Congress on Genetics Applied To Livestock Production* (2014).

[33] Carillier C (2013). A first step toward genomic selection in the multi-breed French dairy goat population. Journal of Dairy Science.

[34] Lecraw, D., Eddleston, P. & McMahon, A. A Value Chain Analysis of the Mongolia Cashmere Industry. *Report prepared for USAID’s Accelerating Sustainable Agriculture Program* (2005).

[35] de Weijer, F. Cashmere Value Chain Analysis Afghanistan. Report prepared for USAID’s Accelerating Sustainable Agriculture Program (2007).

[36] Mcgregor, B. A. Australian cashmere: attributes and processing. *Rural Industries Research and Development Corporation* (2002).

[37] Wang Z (2013). Estimation of genetic parameters for fleece traits in yearling Inner Mongolia Cashmere goats. Small Ruminant Research.

[38] Daniel A (2011). Analyzing and minimizing PCR amplification bias in Illumina sequencing libraries. Genome Biology.

[39] Quail MA (2008). A large genome center’s improvements to the Illumina sequencing system. Nature Methods.

[40] Nakamura K (2011). Sequence-specific error profile of Illumina sequencers. Nucleic Acids Research.

[41] Zhang Y (2014). Estimates of genetic parameters and genetic changes for fleece traits in Inner Mongolia cashmere goats. Small Ruminant Research.

[42] Goodale, H. D. Dominant vs. Non-Dominant Genes: In the Multiple Factor Hypothesis of Size Inheritance. *Journal of Heredity* (1932).

[43] Wei J (2015). The transcriptome research progresses of skin hair follicle development. Hereditas.

[44] Lashmar SF, Visser C, Van Marle-Köster E (2015). Validation of the 50k Illumina goat SNP chip in the South African Angora goat. South African Journal of Animal Science.

[45] Becker D (2015). The brown coat colour of Coppernecked goats is associated with a non-synonymous variant at theTYRP1locus on chromosome 8. Animal Genetics.

[46] Martin PM, Palhière I, Ricard A, TosserKlopp G, Rupp R (2016). Genome Wide Association Study Identifies New Loci Associated with Undesired Coat Color Phenotypes in Saanen Goats. PLoS ONE.

[47] JW K (2012). Genome-Wide Analysis of the World’s Sheep Breeds Reveals High Levels of Historic Mixture and Strong Recent Selection. PLoS Biology.

[48] Lv FH (2014). Adaptations to climate-mediated selective pressures in sheep. Molecular Biology & Evolution.

[49] Dipoï N (2005). Epithelium-mesenchyme interactions control the activity of peroxisome proliferator-activated receptor beta/delta during hair follicle development. Molecular & Cellular Biology.

[50] Yang ZZ (2005). Dosage-Dependent Effects of Akt1/Protein Kinase Bα (PKBα) and Akt3/PKBγ on Thymus, Skin, and Cardiovascular and Nervous System Development in Mice. Molecular & Cellular Biology.

[51] Mauro TM (2009). Akt2 and SGK3 are both determinants of postnatal hair follicle development. Faseb Journal Official Publication of the Federation of American Societies for Experimental Biology.

[52] Kayserili H (2009). ALX4 dysfunction disrupts craniofacial and epidermal development. Human Molecular Genetics.

[53] Kratochwil K, Dull M, Farinas I, Galceran J, Grosschedl R (1996). Lef1 expression is activated by BMP-4 and regulates inductive tissue interactions in tooth and hair development. Genes & Development.

[54] Petersson M (2011). TCF/Lef1 activity controls establishment of diverse stem and progenitor cell compartments in mouse epidermis. Embo Journal.

[55] Boras K, Hamel PA (2002). Alx4 binding to LEF-1 regulates N-CAM promoter activity. Journal of Biological Chemistry.

[56] Gallego MI, Beachy PA, Hennighausen L, Robinson GW (2002). Differential requirements for shh in mammary tissue and hair follicle morphogenesis. Developmental Biology.

[57] Ellis TSI (2003). Overexpression of Sonic Hedgehog suppresses embryonic hair follicle morphogenesis. Developmental Biology.

[58] Botchkarev VA (1998). A New Role for Neurotrophin-3: Involvement in the Regulation of Hair Follicle Regression (Catagen). American Journal of Pathology.

[59] Baldeck N (2015). FF483–484 motif of human Polη mediates its interaction with the POLD2 subunit of Polδ and contributes to DNA damage tolerance. Nucleic Acids Research.

[60] Park D, Jeong HO, Kim BC, Ha YM, Chung HY (2011). Computational Approach to Identify Enzymes That Are Potential Therapeutic Candidates for Psoriasis. Enzyme Research.

[61] Schumacher M (2014). Efficient keratinocyte differentiation strictly depends on JNK-induced soluble factors in fibroblasts. Journal of Investigative Dermatology.

[62] Denda S (2012). Ryanodine receptors are expressed in epidermal keratinocytes and associated with keratinocyte differentiation and epidermal permeability barrier homeostasis. Journal of Investigative Dermatology.

[63] Wang X (2013). Effects of TRAP-1-Like Protein (TLP) Gene on Collagen Synthesis Induced by TGF-β/Smad Signaling in Human Dermal Fibroblasts. PLoS ONE.

[64] Lin CE, Kaptein JS, Sheikh J (2017). Differential expression of microRNAs and their possible roles in patients with chronic idiopathic urticaria and active hives. Allergy & Rhinology.

[65] Du X (2014). An update of the goat genome assembly using dense radiation hybrid maps allows detailed analysis of evolutionary rearrangements in Bovidae. BMC Genomics.

[66] Wu YP (2009). A fine map for maternal lineage analysis by mitochondrial hypervariable region in 12 Chinese goat breeds. Animal Science Journal.

[67] Li H (2009). The Sequence Alignment/Map format and SAMtools. Bioinformatics.

[68] Mckenna A (2010). The Genome Analysis Toolkit: a MapReduce framework for analyzing next-generation DNA sequencing data. Genome Research.

[69] Matukumalli LK (2009). Development and characterization of a high density SNP genotyping assay for cattle. PloS one.

[70] Pritchard JK, Stephens M, Donnelly P (2000). Inference of population structure using multilocus genotype data. Genetics.

[71] Patterson N, Price AL, Reich D (2006). Population structure and eigenanalysis. PLoS genet.

[72] Kang HM (2010). Variance component model to account for sample structure in genome-wide association studies. Nature genetics.

