# Supplementary material for: Genome-wide Target Enrichment-aided Chip Design: a 66 K SNP Chip for Cashmere Goat
Source: Sci Rep. 2017 Aug 17;7:8621. doi: 10.1038/s41598-017-09285-z (PMC5561203; doi:10.1038/s41598-017-09285-z)
Supplement: Supplementary file 1 — Supplemental Figure [file 41598_2017_9285_MOESM1_ESM.pdf]

Genome-wide Target Enrichment-aided Chip Design: a 66K SNP Chip for Cashmere Goat

Xian Qiao[1#], Rui Su[1,2, 3,4,5#], Yang Wang[6,7#], Ruijun Wang[1,3,4,5#], Ting Yang[6,7], Xiaokai Li[1], Wei Chen[10,17], Shiyang He[7], Yu Jiang[9], Qiwu Xu[6,8], Wenting Wan[11], Yaolei Zhang[6,8], Wenguang Zhang[1], Jiang Chen[6,7], Bin Liu[12], Xin Liu[6, 8], Yixing Fan[1], Duoyuan Chen[7], Huaizhi Jiang[13], Dongming Fang[7], Zhihong Liu[1,3,4,5], Xiaowen Wang[7], Yanjun Zhang[1,3,4,5], Danqing Mao[7], Zhiying Wang[1,3,4,5], Ran Di[14], Qianjun Zhao[14], Tao Zhong[15], Huanming Yang[7,16], Jian Wang[7,16], Wen Wang[5], Yang Dong[7,10,17], Xiaoli Chen[6,7*], Xun Xu[6,7*], Jinquan Li[1,2,3,4*]

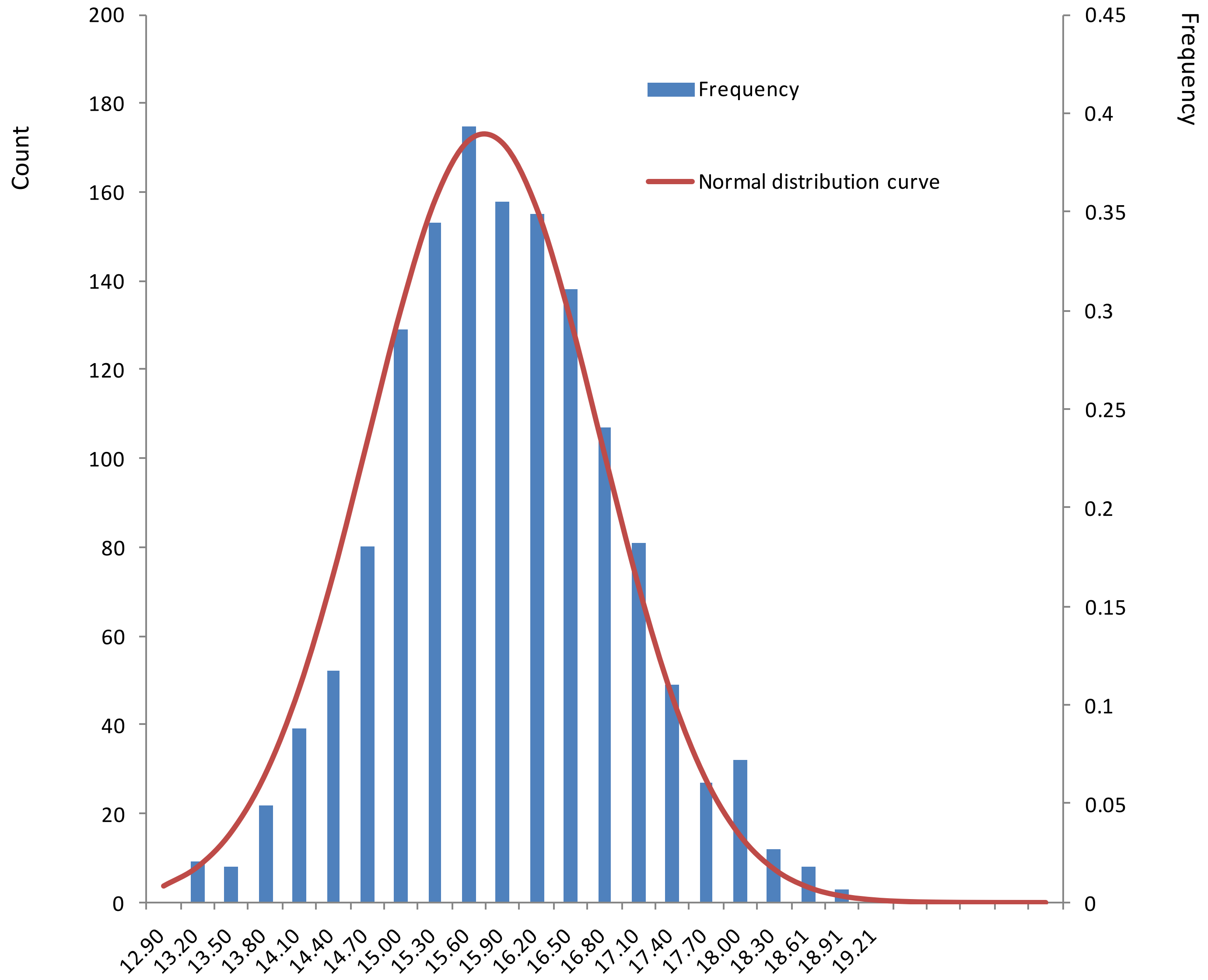
Count
Frequency
Frequency
Normal distribution curve
200
180
160
140
120
100
80
60
40
20
0
0.45
0.4
0.35
0.3
0.25
0.2
0.15
0.1
0.05
0
12.90
13.20
13.50
13.80
14.10
14.40
14.70
15.00
15.30
15.60
15.90
16.20
16.50
16.80
17.10
17.40
17.70
18.00
18.30
18.61
18.91
19.21
